# Extracorporeal life support in COVID‐19‐related acute respiratory distress syndrome: A EuroELSO international survey

**DOI:** 10.1111/aor.13940

**Published:** 2021-03-28

**Authors:** Sebastian Mang, Armin Kalenka, Lars Mikael Broman, Alexander Supady, Justyna Swol, Guy Danziger, André Becker, Sabrina I. Hörsch, Thilo Mertke, Ralf Kaiser, Hendrik Bracht, Viviane Zotzmann, Frederik Seiler, Robert Bals, Fabio Silvio Taccone, Onnen Moerer, Roberto Lorusso, Jan Bělohlávek, Ralf M. Muellenbach, Philipp M. Lepper, for the COVEC‐Study Group, Nicholas Barrett, Daniel Duerschmied, Eddy Fan, Falk Fichtner, Hendrik Haake, Frank Langer, Haitham Mutlak, Markus Kredel, Thomas Müller, Alessandro Protti, Alexander Raddatz, Tobias Spangenberg, Dawid Staudacher, Holger Wehrfritz, Tobias Wengenmayer, Arne Westheider, Simon Dang Van, Cedric Daubin, Philippe Gaudard, Thomas Godet, Pierre‐Grégoire Guinot, Loïc Le Guennec, Bruno Megarbane, Alain Mercat, Romain Sonneville, Elie Zogheib, Tai Pham, Hadrien Winiszewski, Peter Schellongowski, Thomas Staudinger, Dominik Wiedemann, Corinna Velik‐Salchner, Michael Joannidis, Marc Bodenstein, Heinrich Volker Groesdonk, Stefan Guth, Matthias Hecker, Faeq Husain‐Syed, Christian Jung, L. Christian Napp, Ruslan Natanov, Georg Trummer, Sascha Treskatsch, Henryk Welp, Leonello Avalli, Lorenzo Ball, Mirko Belliato, Manuela Bonizzoli, Emma Borrelli, Giacomo Cavallaro, Andrea Franci, Silvia Gramaticopolo, Mauro Panigada, Luigi Tritapepe, Salman Abdulaziz, David Bracco, Yiorgos Alexandros, Ari Joffe, A. Dave Nagpal, Ying Sia, Georg Auzinger, Vasileios Zochios, Alejandro Garcia, Katja Gist, Dana Lustbader, Demetris Yannopoulos, R. Scott Stephens, Joseph Tonna, Linda Paxton, Hitoshi Hirose, Bo Kim, Magnus Dalén, Martin Balik, David Janak, Luis Castillo, Alejandro Bruhn, Jorge Luis Alvarado Socarras, Taeyun Kim, Hyoung Soo Kim, Joung Hun Byun, Guilherme Mainardi, Pedro Mendes, Raphaël Giraud, Philip Fortuna, Tatsuma Fukuda, Jacinta Maas, Dariusz Maciejewski, Deblal Pandit, Yosv Psz, Peter Radsel, Gangfeng Yan

**Affiliations:** ^1^ Interdisciplinary COVID‐19‐Center University Medical Centre, Saarland University Homburg Germany; ^2^ Department of Internal Medicine V—Pneumology, Allergology and Critical Care Medicine University Medical Centre, Saarland University Homburg Germany; ^3^ Department of Anaesthesiology and Intensive Care Medicine District Hospital Bergstrasse University Hospital Heidelberg Heppenheim Germany; ^4^ ECLS Centre Karolinska, Department of Pediatric Perioperative Medicine and Intensive Care Karolinska University Hospital Stockholm Sweden; ^5^ Department of Medicine III (Interdisciplinary Medical Intensive Care), Medical Center—University of Freiburg, Faculty of Medicine University of Freiburg Freiburg Germany; ^6^ Department of Pneumology, Allergology and Sleep Medicine, and Intensive Care Medicine Paracelsus Medical University, General Hospital Nuremberg Nuremberg Germany; ^7^ Department of Anaesthesiology Critical Care Medicine and Pain Medicine University Medical Centre Saarland University Homburg Germany; ^8^ Department of Anaesthesiology and Critical Care Medicine University Hospital of Ulm Ulm Germany; ^9^ Department of Intensive Care Erasme University Hospital Université Libre de Bruxelles Brussels Belgium; ^10^ Department of Anaesthesiology University Hospital of Göttingen Göttingen Germany; ^11^ Cardio‐Thoracic Surgery Department—Heart & Vascular Centre Maastricht University Medical Centre Maastricht The Netherlands; ^12^ 2nd Department of Internal Cardiovascular Medicine General University Hospital Prague Czech Republic; ^13^ Department of Anaesthesiology and Critical Care Medicine Campus Kassel of the University of Southampton Kassel Germany

**Keywords:** COVID‐19, COVID‐19‐induced acute respiratory distress syndrome, extracorporeal membrane oxygenation, extracorporeal life support, SARS‐CoV‐2, survey

## Abstract

Extracorporeal life support (ECLS) is a means to support patients with acute respiratory failure. Initially, recommendations to treat severe cases of pandemic coronavirus disease 2019 (COVID‐19) with ECLS have been restrained. In the meantime, ECLS has been shown to produce similar outcomes in patients with severe COVID‐19 compared to existing data on ARDS mortality. We performed an international email survey to assess how ECLS providers worldwide have previously used ECLS during the treatment of critically ill patients with COVID‐19. A questionnaire with 45 questions (covering, e.g., indication, technical aspects, benefit, and reasons for treatment discontinuation), mostly multiple choice, was distributed by email to ECLS centers. The survey was approved by the European branch of the Extracorporeal Life Support Organization (ELSO); 276 ECMO professionals from 98 centers in 30 different countries on four continents reported that they employed ECMO for very severe COVID‐19 cases, mostly in veno‐venous configuration (87%). The most common reason to establish ECLS was isolated hypoxemic respiratory failure (50%), followed by a combination of hypoxemia and hypercapnia (39%). Only a small fraction of patients required veno‐arterial cannulation due to heart failure (3%). Time on ECLS varied between less than 2 and more than 4 weeks. The main reason to discontinue ECLS treatment prior to patient’s recovery was lack of clinical improvement (53%), followed by major bleeding, mostly intracranially (13%). Only 4% of respondents reported that triage situations, lack of staff or lack of oxygenators, were responsible for discontinuation of ECLS support. Most ECLS physicians (51%, IQR 30%) agreed that patients with COVID‐19‐induced ARDS (CARDS) benefitted from ECLS. Overall mortality of COVID‐19 patients on ECLS was estimated to be about 55%. ECLS has been utilized successfully during the COVID‐19 pandemic to stabilize CARDS patients in hypoxemic or hypercapnic lung failure. Age and multimorbidity limited the use of ECLS. Triage situations were rarely a concern. ECLS providers stated that patients with severe COVID‐19 benefitted from ECLS.

## Graphical abstract

276 ECMO professionals from 98 centers worldwide participated in this EuroELSO‐associated online survey investigating the use of ECMO for severe COVID‐19. Extracorporeal life support (ECLS) for severe COVID‐19 was used predominantly in veno‐venous configuration (87%) to treat isolated hypoxic respiratory failure (50%). Age and multimorbidity limited the use of ECLS. Overall mortality of COVID‐19 patients on ECLS was estimated to be about 55%.

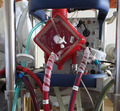

## 1 INTRODUCTION

Early in 2020, countries worldwide have been facing a surge of patients with acute respiratory distress syndrome (ARDS) due to pandemic Severe Acute Respiratory Syndrome Coronavirus‐2 (SARS‐CoV‐2) disease 2019 (COVID‐19). Survival of those most severely affected by COVID‐19‐related ARDS (CARDS) might depend on extracorporeal membrane oxygenation (ECLS) as bridge to recovery.^1^, ^2^, ^3^

In this global pandemic, hospitals and healthcare systems have been pushed to the verge of collapse. During the first phase of the COVID‐19 pandemic, the number of critically ill patients requiring invasive ventilation often exceeded ventilator capacities, creating a need for intensive care unit (ICU) triage.^4^ In this scenario, it was highly unlikely that ECLS would be broadly recommended to critical care providers to treat COVID‐19, given its high demands on personnel and resources.^5^ In its initial guidance document, ELSO considered to offer ECLS only to specific patients not responding to maximal conventional therapy, including proning and neuromuscular blockade.^6^ Additionally, early reports suggested mortality rates could be higher than 90% in COVID‐19 patients supported with ECLS.^7^

A recent trial reported that veno‐venous (VV‐)ECLS reduced 60‐day mortality in non‐COVID‐19‐related ARDS to 35% in the ECLS group versus 46% in the conventional management group (relative risk 0.76, 95% CI 0.55–1.04; *P* = .09).^8^ The study highlighted that VV‐ECLS can facilitate protective ventilation of ARDS patients with reduced tidal volumes, plateau, and driving pressures,^9^ mostly due to effective extracorporeal CO_2_ removal. CARDS might not differ as much from non‐COVID ARDS as was previously expected.^10^ Physiological considerations make it thus reasonable to think about ECLS as a bail‐out strategy in critically ill patients with CARDS. A recently published retrospective data suggested that mortality of patients with CARDS receiving VV‐ECLS might be comparable to past ARDS cohorts.^11^ Given that COVID‐19 pathophysiology is still poorly understood, little is currently known about how to tailor ECLS treatment to meet COVID‐19 specific challenges, for example, hypercoagulable state^12^ or how long ECLS should be continued when patients fail to improve. We therefore designed an online survey to elicit how ECLS providers worldwide have previously employed ECLS to treat critically ill COVID‐19 patients. Our survey was approved by the European branch of the Extracorporeal Life Support Organization (EuroELSO).

## 2 METHODS

We created a questionnaire consisting of 45 questions and distributed it to 4193 physicians that had published on an ECLS‐related topic since the year 2000 in a PubMed‐listed journal with an available e‐mail using a commercially available internet survey platform (SurveyMonkey Inc., San Mateo, CA, USA).

The ethical committee (Ärztekammer des Saarlandes) waived the need for a formal approval since the questionnaire did not retrieve actual patient data.

## 3 QUESTIONNAIRE

The questionnaire was composed of two sections: The first dealt with general questions regarding contact information, details on hospital and ICU capacity, as well as years of ECLS experience. The second part was designed to elicit most common indications for ECLS use in COVID‐19, details about ECLS circuit configuration as well as complications and reasons for possible treatment discontinuation. We did not ask for any patient‐specific data. For conformity reasons and to facilitate participation in the survey, most of the questions were multiple choice with two to nine possible answers per question. The last eight items requested the participant to express his extent of agreement with a specific statement about ECLS therapy in the context of COVID‐19 on a visual analog scale. The survey is partly available in the Supporting Information.

The survey questions and multiple‐choice responses with their respective organization in the different sections were circulated and consented between a group of 23 very experienced physicians in this field. When consensus of all questions and answers was reached, the survey was transferred to an online platform (SurveyMonkey Inc.). Automatic data retrieval and descriptive statistics were retrieved through this platform. More than one answer from centers was possible. This was allowed, as many centers comprise several departments with physicians from different backgrounds (e.g., anesthesiology and surgery).

Results from multiple‐choice questions are expressed in median, participants’ extent of agreement or disagreement in mean, and standard deviation in percent. The survey was launched on June 8, 2020; deadline for return was June 20, 2020. Final analysis of results was performed using an extrapolation tool provided by SurveyMonkey as well as SPSS.

Participants were given the opportunity to be listed as collaborators. Those participants who did not supply hospital or contact information or who did not complete the survey could not be included in the list of collaborators.

## 4 RESULTS

### 4.1 General data on ECLS centers and treatment capacities

Two hundred seventy‐six ECLS professionals from 98 centers in 30 different countries on five continents (North America, South America, Europe, Asia, and Australia) responded to the survey. The overall response rate of individual ECLS physicians was 9.0%. Sixty‐four percent of responding centers were ELSO members. Response rate was heavily skewed depending on geography. Seventy‐one percent of the largest participating ECLS centers (more than one hundred COVID‐19 patients treated on the ICU) were located in Europe, 21% in North America. As it was possible to skip questions, sometimes the denominator is less than 276. In this case, the number of respondents is given in brackets.

Centers’ median number of years with ECLS experience was 14, mainly in ECLS treatment of adults or adult and pediatric patients (85%). Only 1.3% of participants were exclusively specialized in neonatal ECLS. Most common numbers of patients supported with ECLS per year prior to COVID‐19 in the participants’ centers ranged from 21 to 50, 13% of centers having even supported more the 100 patients on VV‐ECLS per year prior to the pandemic.

### 4.2 Numbers of patients with COVID‐19 with or without ECLS

The majority of ECLS providers (30%) stated that 2–6 patients with COVID‐19 had received ECLS in their center, with 85% of all centers having supported a maximum of 15 patients on ECLS by survey deadline. ECLS treatment had mostly been initiated in the participants’ hospitals (63%), only a minority of patients was retrieved on ECLS by mobile ECLS retrieval teams from other hospitals.

### 4.3 Indication for ECLS and circuit configuration

**FIGURE 1:**
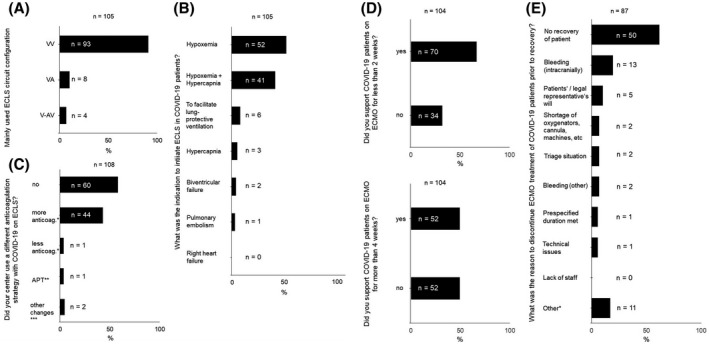
A–C, Extracorporeal life support (ECLS) circuit configuration, indication, and anticoagulation strategy. *measured in higher or reduced prothrombin time (PTT) or **activated clotting time (ACT); ***were specified as direct thrombin inhibition (*n* = 1) and Anti‐Xa‐activity (*n* = 1). (D,E) Duration of ECLS therapy and reasons for treatment discontinuation. *were specified as futility (*n* = 2), intractable septic shock (*n* = 1), multi‐organ failure (*n* = 3), and bleeding other than intracranial (*n* = 1)

The most common reason to initiate ECLS for COVID‐19 was isolated hypoxemia (50%), followed by a combination of hypoxemia and hypercapnia (39%). Isolated hypercapnia was rarely a reason to cannulate a patient (3%). Only 6% stated that ECLS was started to facilitate lung‐protective ventilation (*n* = 105). The majority of ECLS cannulations (88%) were performed in VV configuration. Eight percent of centers used veno‐arterial configuration (VA‐ECLS) in one or more patients, and 3% had to extend to a V‐AV circuit in at least one case (one venous draining cannula, one arterial returning cannula, and one venous returning cannula). In those cases, where an arterial cannulation was required, the indications were specified as biventricular failure (*n* = 2) and, in one case, right heart failure due to pulmonary embolism (*n* = 1). See also Figure 1A,B.

### 4.4 Anticoagulation management

Targeting anticoagulation therapy in patients with COVID‐19 on ECLS, 60% of participants (*n* = 110) stated that they did not change their standard anticoagulation strategy compared to cases of ARDS due to other causes. Forty percent used higher doses of anticoagulants than usual, monitored by higher prothrombin time or higher activated clotting time. Only one of 110 ECLS providers stated that they deliberately used lower doses of anticoagulants than usual for ECLS in COVID‐19. Antiplatelet therapy was also rarely used (1%) to prevent clotting. The details of the anticoagulants or antiplatelet agents administered were not part of the survey. See also Figure 1C.

### 4.5 Reasons to abstain from ECLS

The two main reasons to refrain from ECLS initiation were patient age (74%) and comorbidities (85%, not further specified). Twenty‐eight percent of participants stated that ECLS was withdrawn due to a patient’s known or suspected wishes. Nine percent decided against ECLS because it was not actively recommended for COVID‐19‐induced ARDS by responsible scientific societies at that time. Seven percent reported that a surge of COVID‐19 patients and overwhelming workload made ECLS impracticable. Only 5% of participants reported that they had to abstain from ECLS initiation due to a shortage of oxygenators, machines, or ECLS cannulas.

### 4.6 Duration of ECLS support

Most patients were supported with ECLS for less than 2 weeks. However, 50% of all participants stated that they had also treated patients with ECLS for more than 4 weeks (Figure 1D).

### 4.7 Reasons for ECLS discontinuation

Seventy‐two percent of participants confirmed that their center would withdraw ECLS if there was no perspective for a COVID‐19 patient to recover. If ECLS treatment was discontinued prior to recovery, futility was mostly stated as the reason (*n* = 50 from 94 responses, 53%). ECLS‐related complications were the second most important reason for treatment discontinuation. Fourteen percent of ECLS providers stated that they had terminated ECLS due to major bleeding (*n* = 15), mainly intracranial hemorrhage (*n* = 13) and, less frequently, extracranially. In 1% of cases, further unspecified technical issues led to ECLS withdrawal. The question also offered “lack of staff” as a possible answer, which was not chosen. However, 2% of participants (*n* = 2, Germany and France) stated that a triage situation forced physicians to discontinue ECLS prior to the patient’s possible recovery. Two percent of respondents named lack of ECLS oxygenators, ECLS machines, or consumables as the reason for ECLS discontinuation. See also Figure 1E.

### 4.8 Estimation of patients’ outcome

When asked to estimate the percentage of patients who died while on ECLS due to COVID‐19, average mortality was estimated to be 55%, meaning that 45% of patients had survived on ECLS at least until the end of the survey.

### 4.9 ECLS providers’ opinions on COVID‐19 and ECLS

**FIGURE 2:**
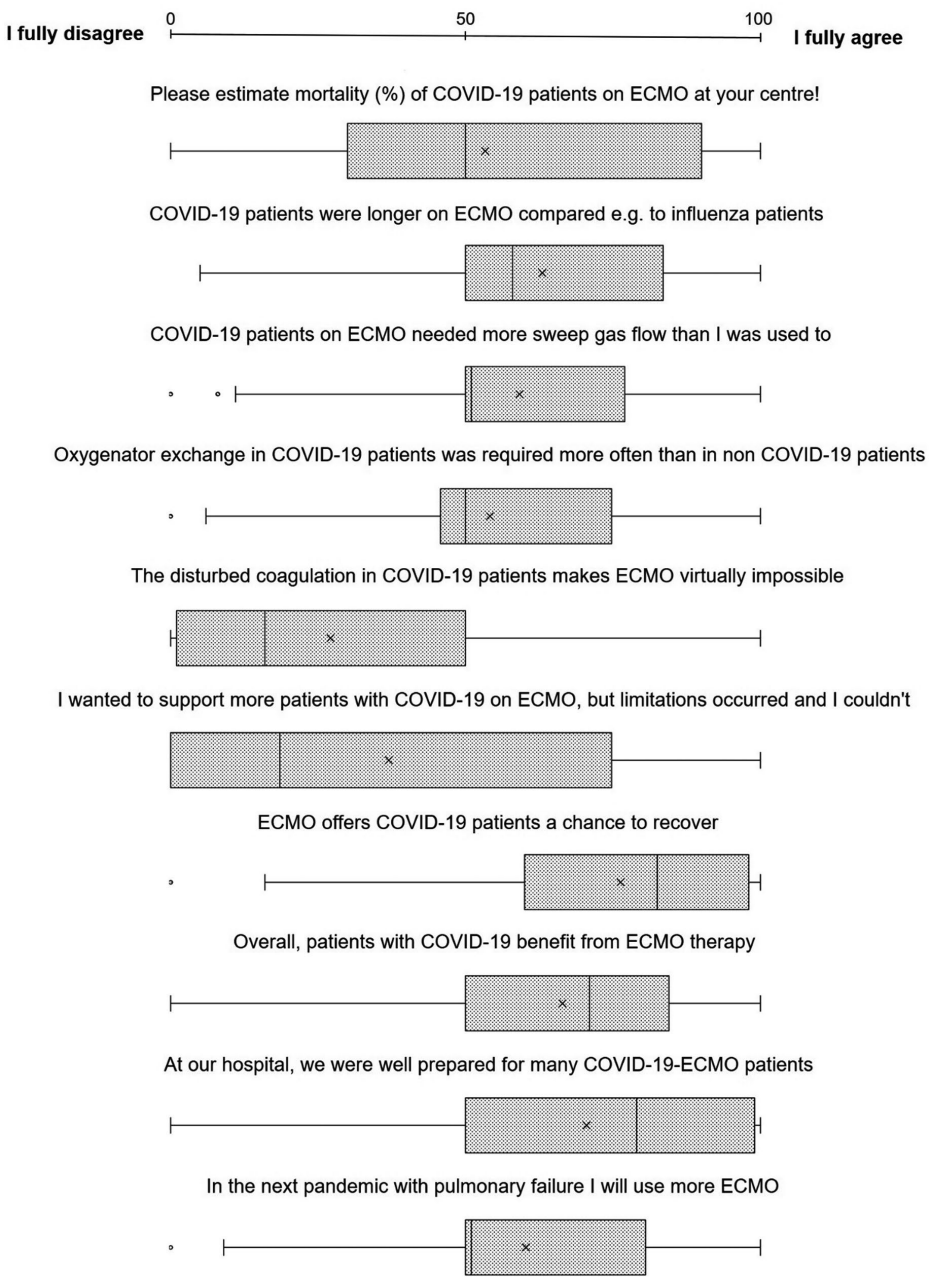
Participants’ extent of agreement to statements about COVID‐19 and extracorporeal life support (ECLS) therapy; 0 = full disagreement, 100% = full agreement. Results are expressed in box plots. The left box barrier equals the 25th percentile, and the right barrier equals the 75th percentile. Median is expressed by the full line inside the box, and mean is marked as an “x”

The last eight questions were designed to investigate a participant’s opinion on certain statements about ECLS and COVID‐19, measured in percentage of agreement. Participants agreed to 58% (IQR 33%) on median that patients were longer on ECLS due to COVID‐19 compared to other causes of ARDS. The claim that CARDS patients on ECLS required more sweep gas flow than what the individual ECLS physician was used to was accepted by 58% (IQR 26.8%). The statement that oxygenator change was more frequently required in CARDS patients on ECLS had an acceptance rate of 50% on average (IQR 27%). The claim that disturbed coagulation in COVID‐19 patients would make ECLS impossible was mostly rejected (16% agreement, IQR 48%). The assumption that ECLS offers patients with COVID‐19‐induced ARDS a chance to recover found relatively strong acceptance (agreement extent of 82%, IQR 38%), with 71% (IQR 34%) agreement with the claim that CARDS patients benefitted from ECLS therapy. Box plots are displayed in Figure 2.

For results not outlined here, see Supporting Information.

## 5 DISCUSSION AND CONCLUSIONS

Survey results prove that critical care providers worldwide have repeatedly successfully used ECLS to support patients in COVID‐19‐related respiratory failure despite official recommendations and were limited to carefully singled‐out patients. At the time the survey was launched, data regarding the outcome of COVID‐19 patients treated with ECLS were limited to case reports and small case series. Recent retrospective cohort studies on COVID‐19 patients receiving ECLS have produced encouraging results^11^ but were unpublished at that time. Critical care providers hence had to rely on what was already known about ARDS from different causes. Both clinical experience and pathophysiology of CARDS made ECLS appealing to treat COVID‐19, even when data on mortality or complication rate were missing.

Several groups have reported evidence that COVID‐19 might be associated with a hypercoagulable state, resulting in an increased risk of thromboembolic complications.^12^, ^13^ Exposing hypercoagulable blood to the artificial surface of an ECLS circuit could therefore come with a considerable risk of thromboembolism. Interestingly, when asked about ECLS‐related complications in COVID‐19, 13% of ECLS providers stated that ECLS had to be terminated due to major bleeding, but only one case of relevant pulmonary embolism on VV‐ECLS was reported. It should be noted though that minor thrombosis or thromboembolism could have remained undetected during ECLS therapy; hence, a reliable incidence of all thrombotic events cannot be provided or was underestimated by the ECLS providers. Nevertheless, given that 50% of all participants had treated patients on ECLS for more than 4 weeks, the overall occurrence of lethal thromboembolism on ECLS in COVID‐19 was surprisingly low. Only a minority of ECLS physicians said that they increased the dosage of anticoagulants to prevent clotting on ECLS. There was also no general agreement that oxygenator change was required more frequently compared to patients with ARDS from other causes. Although increased rate of oxygenator pump head thrombosis in COVID‐19 has been reported,^14^ suspected hypercoagulation in COVID‐19 did not seem to translate to a higher incidence of life‐threatening pulmonary or systemic thromboembolism in patients with COVID‐19 on ECLS. Prospective studies should address how much anticoagulation for ECLS in COVID‐19 is needed so that both the risk of thrombosis and hemorrhage can be minimized.

Although predominantly affecting the lungs, multiple organ involvement of COVID‐19 has been reported. Cardiac complications in COVID‐19 have recently gathered broad attention. Acute myocardial injury in the absence of macroscopic coronary artery disease is suspected to occur in up to 10% of patients especially in the critically ill.^15^, ^16^, ^17^, ^18^ In a recent retrospective analysis, Zayat et al found that, among others, increased levels of amino‐terminal pro‐brain natriuretic peptide (NT‐pro‐BNP) predict worse survival in COVID‐19 patients on extracorporeal life support.^19^ While 11% of our participants reported that they employed also veno‐arterial ECLS for COVID‐19, only 3% of participants specified that arterial cannulation was required because of acute heart or circulatory failure, in one case caused by fulminant pulmonary embolism. So far, our survey results do not support the hypothesis that severe cardiac involvement in COVID‐19 translated to an increased need for cardiac support (e.g., with VA‐ECLS) in patients with CARDS.

A recently published study investigating outcomes of all adult patients with CARDS treated with ECLS using a EuroELSO registry estimated overall 90‐day mortality to be less than 40%.^11^ When asked about the mortality of COVID‐19 patients on ECLS, the mean estimation in our survey was 55%, meaning that roughly 45% could be weaned off extracorporeal support at survey deadline. If these data translate to real‐life mortality, survival of COVID‐19 patients on ECLS could be comparable to non‐COVID‐19‐induced ARDS. At the time of report, no prospective data on the outcome nor complication rate of ECLS therapy in COVID‐19 are available. For the time being, our survey suggests that ECLS for COVID‐19 is practicable, effective, and does not lead to higher complication rates in COVID‐19 if it is utilized in experienced ECLS centers. Most participants agreed that their patients benefitted from ECLS therapy and stated that, in a hypothetical scenario of a future respiratory pandemic, they would use it more readily to treat acute respiratory failure.

Our survey has several limitations. Firstly, while it reflects opinions of a fairly large cohort of physicians providing ECLS services, we nevertheless did not ask for patient‐specific data outcome. Hence, we cannot provide statistical evidence on ECLS‐related end points, for example, overall survival, 28‐day mortality, or discharge from ICU. Secondly, the survey was addressed mainly to tertiary care centers who have repeatedly published on ECLS, leading to a skewness of representation, possibly to the disadvantage of primary and secondary care hospitals with less ECLS experience.

Critical care providers need to know not only whether to use ECLS but also how to manage ECLS therapy in a disease as poorly understood as COVID‐19. Currently, this knowledge is expanding.^11^ Across Europe, more than 2100 patients in 180 centers were already treated on ECLS for respiratory and/or circulatory failure due to COVID‐19 (https://www.euroelso.net/covid‐19/covid‐19‐survey/; last accessed on November 23). The data from this survey (theoretical considerations early in the pandemia) and the EuroELSO (real‐world data with weekly updates) data are in line, suggesting that ECLS is a useful adjunctive tool in COVID‐19‐related respiratory failure. As the survey was conducted in June, a follow‐up survey may be indicated at the appropriate time.

## CONFLICT OF INTEREST

Robert Bals declares funding from AstraZeneca, Boehringer Ingelheim, GlaxoSmithKline, Grifols, Novartis, CLS Behring, the German Federal Ministry of Education and Research (BMBF) Competence Network Asthma and COPD (ASCONET), Sander‐Stiftung, Schwiete‐Stiftung, Krebshilfe and Mukoviszidose eV. Conflicts that the editors consider relevant to the content of the manuscript have been disclosed. All other authors declare no potential conflicts of interest.

## AUTHOR CONTRIBUTIONS

S.M., A.K., A.B., G.D., S.H., T.M., and P.L. drafted the survey. L.M.B, J.S., H.B., F.S.T., O.M., R.L., J.B., and R.M. revised the survey for important content. P.L. launched the survey and oversaw collection of data. R.K. provided IT tools for survey preparation. S.M. drafted the manuscript. A.K., L.M.B., A.S., J.S., G.D., S.H., F.S., R.B., F.S.T., O.M., R.L, J.B., R.M., and P.L. revised the manuscript for important intellectual content. All authors have seen and approved the final version of the manuscript.

## CONSENT TO BE LISTED AS COLLABORATOR

All colleagues who participated in the survey and agreed to be named publicly were included as collaborators.

## Supporting information

Supplementary Material

## Appendix

**The COVID‐19 ECLS (COVEC) study group**

Nicholas Barrett, Guy’s and St Thomas’ NHS Foundation Trust, London, UK;

Daniel Duerschmied, University Hospital of Freiburg, Freiburg, Germany;

Eddy Fan, Toronto General Hospital, Toronto, Canada;

Falk Fichtner, Universitätsklinikum Leipzig, Leipzig, Germany;

Hendrik Haake, Krankenhaus Maria Hilf, Mönchengladbach, Germany;

Frank Langer, University Medical Centre, Saarland University, Homburg/Saar, Germany;

Haitham Mutlak, Sana Klinikum Offenbach, Offenbach, Germany;

Markus Kredel, University Hospital of Würzburg, Würzburg, Germany;

Thomas Müller, University Hospital of Regensburg, Regensburg, Germany;

Alessandro Protti, Humanitas Research and Clinical Centre‐IRCCS, Milan, Italy

Alexander Raddatz, University Medical Centre, Saarland University, Homburg/Saar, Germany;

Tobias Spangenberg, Marienkrankenhaus Hamburg, Hamburg, Germany;

Dawid Staudacher, University Hospital of Freiburg, Freiburg, Germany;

Holger Wehrfritz, University Medical Centre, Saarland University, Homburg/Saar, Germany

Tobias Wengenmayer, University Hospital of Freiburg, Freiburg, Germany;

Arne Westheider, University Medical Centre, Saarland University, Homburg/Saar, Germany.

**Survey collaborators**

**France**

Simon Dang Van, University Hospital of Angers, France;

Cedric Daubin, Hospital Center University of Caen Normandie, France;

Philippe Gaudard, CHU Montpellier, University Montpellier, France;

Thomas Godet, University Hospital of Clermont‐Ferrand, France;

Pierre‐Grégoire Guinot, Département d’Anesthésie‐Réanimation, Centre Hospitalier Universitaire de Dijon, France;

Loïc Le Guennec, Hôpital Universitaire Pitié Salpêtrière, Paris, France;

Bruno Megarbane, Lariboisière Hospital, University of Paris, France;

Alain Mercat, Centre Hospitalier Universitaire d'Angers, France;

Romain Sonneville, Bichat‐Claude Bernard University Hospital, France;

Elie Zogheib, Medical Intensive care—CHU Amiens, France;

Tai Pham, Medecine Intensive‐Réanimation, Hôpital Bicêtre, APHP, Le Kremlin Bicêtre, France;

Hadrien Winiszewski, Centre Hospitalier Régional Universitaire de Besançon, France.

**Austria**

Peter Schellongowski, Dept. of Medicine I, Medical University of Vienna, Austria;

Thomas Staudinger, Dept. of Medicine I, Medical University of Vienna, Austria;

Dominik Wiedemann, Department of Cardiac Surgery, Medical University of Vienna, Austria;

Corinna Velik‐Salchner, Medical University Innsbruck, Austria;

Michael Joannidis, Medical University Innsbruck, Dept. Internal Medicine, Division of Intensive Care and Emergency Medicine, Austria.

**Germany**

Marc Bodenstein, University Medical Center Mainz, Germany;

Heinrich Volker Groesdonk, Dept. of Intensive Care Medicine and Intermediate Care; Helios Clinic Erfurt, Germany;

Stefan Guth, Kerckhoff‐Klinik, Bad Nauheim, Germany;

Matthias Hecker, University Hospital Giessen, Medical Clinic II, Germany;

Faeq Husain‐Syed, University Hospital Giessen and Marburg, Giessen, Germany;

Christian Jung, Heinrich‐Heine‐University Düsseldorf, Germany;

L. Christian Napp, Hannover Medical School, Germany;

Ruslan Natanov, Hannover Medical School, Germany;

Georg Trummer, Universitaets‐Herzzentrum Freiburg Bad Krotzingen, Germany;

Sascha Treskatsch, Charité—University Hospital Berlin, Department of Anesthesiology and Intensive Care Medicine, Berlin, Germany;

Henryk Welp, University Hospital Münster, Germany.

**Italy**

Leonello Avalli, Ospedale San Gerardo, Monza, Italy;

Lorenzo Ball, Università degli studi di Genova, Genova, Italy;

Mirko Belliato, Foundation IRCCS Policlinico San Matteo, Pavia, Italy;

Manuela Bonizzoli, Intensive Care Unit, Careggi Hospital, Florence, Italy;

Emma Borrelli, University of Siena, Siena, Italy;

Giacomo Cavallaro, Fondazione IRCCS Ca' Granda Ospedale Maggiore Policlinico, Milano, Italy;

Andrea Franci, Azienda Odpedaliero Universitaria Careggi, Florence, Italy;

Silvia Gramaticopolo, Ospedale San Bortolo Vicenza, Italy;

Mauro Panigada, Fondazione IRCCS Ca' Granda Ospedale Maggiore Policlinico Milano, Italy;

Luigi Tritapepe, San Camillo‐Forlanini Hospital, Rome, Italy.

**Saudi Arabia**

Salman Abdulaziz, King Saud Medical City, Saudi Arabia.

**Canada**

David Bracco, McGill University Health Center, Montreal, Canada;

Yiorgos Alexandros, Cavayas Hôpital du Sacré‐Coeur de Montréal, Montréal, Canada;

Ari Joffe, Stollery Children's Hospital, Alberta, Canada;

A. Dave Nagpal, London Health Sciences Centre, London, Canada;

Ying Sia, University Institute of Cardiology and Respirology of Quebec, Quebec, Canada.

**United Kingdom**

Georg Auzinger, King's College Hospital London, United Kingdom;

Vasileios Zochios, University Hospitals of Leicester NHS Trust, Glenfield Hospital ECMO Unit, Lecester, United Kingdom.

**United States of America**

Alejandro Garcia, Johns Hopkins University, Baltimore, Maryland, United States;

Katja Gist, University of Colorado, Boulder, Colorado, United States;

Dana Lustbader, ProHEALTH an OPTUM Company, New York, New York, United States;

Demetris Yannopoulos, University of Minnesota, Minneapolis, Minnesota, United States;

R. Scott Stephens, Johns Hopkins University, Baltimore, Maryland, United States;

Joseph Tonna, University of Utah, Salt Lake City, Utah, United States;

Linda Paxton, St. Mary's Hospital, Richmond, Virginia, United States;

Hitoshi Hirose, Thomas Jefferson University Hospital, Philadelphia, Pennsylvania, United States;

Bo Kim, Johns Hopkins University, Baltimore, Maryland, United States.

**Sweden**

Magnus Dalén, Karolinska University Hospital and Karolinska Institutet, Sweden.

**Czech Republik**

Martin Balik, Complex Cardiac Center, General University Hospital, Prague, Czech Republic;

David Janak, Charles University of Prague, Department of Cardiovascular Surgery, Prague, Czech Republic.

**Chile**

Luis Castillo, Hospital Barros Luco, Santiago, Chile;

Alejandro Bruhn; Pontificia Universidad Catolica de Chile, Santiago, Chile.

**Colombia**

Jorge Luis Alvarado Socarras, Fundacion Cardiovascular de Colombia, Bucaramanga, Santander, Colombia.

**South Korea**

Taeyun Kim, Seongnam Citizens Medical Center, Seongnam, South Korea;

Hyoung Soo Kim, Hallym University Sacred Heart Hospital, Seoul, South Korea;

Joung Hun Byun, Gyeongsang National University College of Medicine, Changwon, South Korea.

**Brazil**

Guilherme Mainardi, Hospital São Camilo, Brazil;

Pedro Mendes, Hospital das Clinicas de São Paulo HC‐FMUSP, São Paulo, Brazil.

**Switzerland**

Raphaël Giraud, Geneva University Hospitals, Geneva, Switzerland.

**Portugal**

Philip Fortuna, Centro Hospitalar Universitário de Lisboa Central, Lisbon, Portugal.

**Japan**

Tatsuma Fukuda, University of the Ryukyus, Nakagami District, Okinawa, Japan.

**The Netherlands**

Jacinta Maas, Leiden University Medical Center, Leiden, The Netherlands.

**Poland**

Dariusz Maciejewski, Regional Teaching Hospital, Bielsko‐Biała, Poland.

**India**

Deblal Pandit, Medica Superspecialty Hospital Kolkata, Calcutta, India.

**Israel**

Yosv Psz, General Intensive Care Unit, Tel Aviv Sourasky Medical Center, Tel Aviv, Israel.

**Slovenia**

Peter Radsel, University Medical Center Ljubljana, Slovenia.

**China**

Gangfeng Yan, Children's Hospital of Fudan University, China.
