# Supplementary material for: Extracorporeal life support in COVID‐19‐related acute respiratory distress syndrome: A EuroELSO international survey
Source: Artif Organs. 2021 Mar 28;45(5):495–505. doi: 10.1111/aor.13940 (PMC8014805; doi:10.1111/aor.13940)
Supplement: Supplementary file 1 — Supplementary Material [file AOR-45-495-s001.docx]

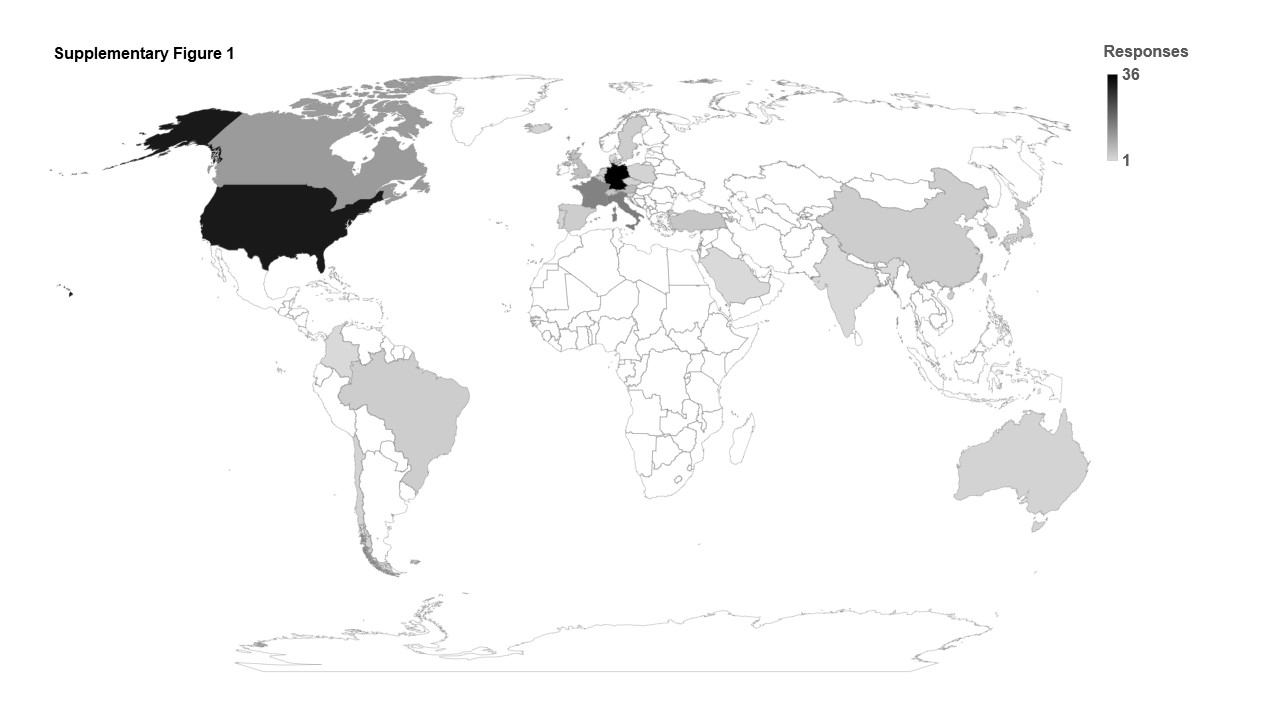


**Supplementary Figure 1:** Geographical distribution of survey responses. 98 centers in 30 different countries on five continents (North America, South America, Europe, Asia, Australia) responded to the survey.

**Supplementary Table 1 – General information about participating ECLS centers**

|  | **Questions** | | | | **Answers** | | | **Number** | **Percentage** |
| --- | --- | --- | --- | --- | --- | --- | --- | --- | --- |
| 1 | What is your hospital’s level of care? | | | | 1. Tertiary Care / University Hospital / Academic 2. Non-Academic 3. General Hospital 4. Community Hospital | | | 176  4  6  1 | 94%  2%  3%  1% |
|  | Responded: **187** | | Skipped: 75 | |  |  |  |  |  |
|  |  | | | |  | | |  |  |
| 2 | How is your institution funded? | | | | 1. Non-government 2. Government 3. mixed | | | 41  109  36 | 22%  59%  19% |
|  | Responded: **186** | | Skipped: 76 | |  |  |  |  |  |
|  |  |  |  |  |  |  |  |  |  |
| 3 | Is your institution an ELSO center? | | | | 1. Yes 2. No | | | 112  63 | 64%  36% |
|  | Responded: **175** | | Skipped: 87 | |  |  |  |  |  |
|  |  |  |  |  |  |  |  |  |  |
| 4 | How many beds does your hospital have in total? | | | | 1. less than 250 2. 250 to 500 3. 501 to 750 4. 751 to 1000 5. more than 1000 | | | 5  33  32  31  74 | 3%  19%  18%  18%  42% |
|  | Responded: **175** | | Skipped: 87 | |  |  |  |  |  |
|  |  |  |  |  |  |  |  |  |  |
| 5 | What is your ICU’s capacity? | | | | 1. 0 to 8 beds 2. 9 to 12 beds 3. 13 to 16 beds 4. 17 to 20 beds 5. 21 to 40 beds 6. more than 40 beds | | | 8  17  25  20  53  53 | 5%  10%  14%  11%  30%  30% |
|  | Responded: **176** | | Skipped: 86 | |  |  |  |  |  |
|  |  |  |  |  |  |  |  |  |  |
| 6 | What is the type of your ICU? | | | | 1. Surgical 2. Non-surgical 3. Interdisciplinary | | | 32  29  116 | 18%  16%  66% |
|  | Responded: **177** | | Skipped: 85 | |  |  |  |  |  |
|  |  |  |  |  |  |  |  |  |  |
| 7 | Did you increase ICU capacity for COVID-19? | | | | 1. Yes 2. No | | | 124  53 | 70%  30% |
|  | Responded: **177** | | Skipped: 85 | |  |  |  |  |  |
|  |  |  |  |  |  |  |  |  |  |
| 8 | By how much did you increase ICU capacity for COVID-19? | | | | 1. Less than 10% 2. 10 to 25% 3. 25 to 50% 4. 50 to 75% 5. 75 to 100% 6. more than 100% | | | 5  16  37  22  17  23 | 4%  13.5%  31%  18.5%  14%  19% |
|  | Responded: **120** | | Skipped: 142 | |  |  |  |  |  |
|  |  |  |  |  |  |  |  |  |  |
| 9 | Did you increase ECLS capacity for COVID-19? | | | | 1. Yes 2. No | | | 78  94 | 45%  55% |
|  | Responded: **172** | | Skipped: 90 | |  |  |  |  |  |
|  |  |  |  |  |  |  |  |  |  |
| 10 | By how much did you increase ECLS capacity for COVID-19? | | | | 1. less than 10% 2. 10 to 25% 3. 25 to 50% 4. 50 to 75% 5. 75 to 100% 6. more than 100% | | | 6  18  20  9  8  15 | 8%  23.5%  26%  12%  10.5%  20% |
|  | Responded: **76** | | Skipped: 186 | |  |  |  |  |  |
|  |  |  |  |  |  |  |  |  |  |

**ECLS** – Extracorporeal membrane oxygenation / **EuroELSO** – European Extracorporeal Life Support Organization / ICU – Intensive care unit / COVID-19 – Coronavirus disease 2019

**Supplementary Table 2 – ECLS centers’ experience with COVID-19 and ECLS**

|  | **Questions** | | | | **Answers** | | | **Number** | **Percentage** |
| --- | --- | --- | --- | --- | --- | --- | --- | --- | --- |
| 1 | How many patients with COVID-19 have been treated in your hospital in total up to now?* | | | | 1. up to 8 2. 9 to 20 3. 21 to 40 4. 41 to 60 5. 61 to 80 6. 81 to 100 7. more than 100 | | | 17  18  15  11  4  9  73 | 11.5%  12%  10%  7.5%  3%  6%  50% |
|  | Responded: **147** | | Skipped: 115 | |  |  |  |  |  |
|  |  | | | |  | | |  |  |
| 2 | How many of these COVID-19 patients needed intensive care? | | | | 1. 1 2. 2 to 6 3. 7 to 10 4. 11 to 15 5. 16 to 20 6. 21 to 50 7. 51 and more | | | 10  17  12  9  11  26  60 | 7%  12%  8%  6%  8%  18%  41% |
|  | Responded: **145** | | Skipped: 117 | |  |  |  |  |  |
|  |  |  |  |  |  |  |  |  |  |
| 3 | How many of these COVID-19 patients have been or are still on ECLS?* | | | | 1. 1 2. 2 to 6 3. 7 to 10 4. 11 to 15 5. 16 to 20 6. 21 to 50 7. 51 to 70 8. 71 and more | | | 26  35  20  18  5  11  0  1 | 22%  30%  17%  15.5%  4%  9.5%  0%  1% |
|  | Responded: **116** | | Skipped: 146 | |  |  |  |  |  |
|  |  |  |  |  |  |  |  |  |  |
| 4 | Where was ECLS initiated in patients with COVID-19 treated at your center? | | | | 1. In my own hospital 2. In another hospital (e.g. community hospital) 3. Both, in own hospital and externally | | | 75  4  41 | 63%  3%  34% |
|  | Responded: **120** | | Skipped: 142 | |  |  |  |  |  |
|  |  |  |  |  |  |  |  |  |  |

***Survey deadline was 20^th^ June, 2020**

**ECLS** – Extracorporeal membrane oxygenation / ICU – Intensive care unit / COVID-19 – Coronavirus disease 2019

**Supplementary Table 3 – Details on ECLS initiation and treatment for COVID-19**

|  | **Questions** | | | | **Answers** | | | **Number** | **Percentage** |
| --- | --- | --- | --- | --- | --- | --- | --- | --- | --- |
| 1 | What was the indication to initiate ECLS support in COVID-19 patients? | | | | 1. Hypoxemia 2. Hypercapnia 3. Combination of hypoxemia / hypercapnia 4. To facilitate lung-protective ventilation 5. Right heart failure 6. Biventricular failure 7. Pulmonary embolism | | | 52  3  41  6  0  2  1 | 49.5%  3%  39%  5.5%  0%  2%  1% |
|  | Responded: **105** | | Skipped: 157 | |  |  |  |  |  |
|  |  | | | |  | | |  |  |
| 2 | What was the main ECLS circuit configuration in COVID-19 patients?  ***VV*** *– veno-venous;* ***VA*** *– veno-arterial;* ***V-VA*** *– veno-venous-arterial (1 venous draining cannula; 1 venous returning cannula, 1 arterial returning cannula*) | | | | 1. VV 2. VA 3. V-VA 4. Other* | | | 93  8  4*  2* | 87.5%  7.5%  3%  2% |
|  | Responded: **106** | | Skipped: 156 | |  |  |  |  |  |
|  |  |  |  |  |  |  |  |  |  |
| 3 | Did your center use a different anticoagulation strategy in patients with COVID-19 on ECLS compared to other patients on ECLS? | | | | 1. no 2. more anticoagulation** 3. less anticoagulation** 4. inhibition of platelet aggregation 5. other changes in anticoagulation management | | | 60  44  1  1  2 | 55.5%  40.5%  1%  1%  2% |
|  | Responded: **108** | | Skipped: 152 | |  |  |  |  |  |
|  |  |  |  |  |  |  |  |  |  |
| 4 | State reasons why patients with COVID-19 were not offered ECLS support despite qualification (multiple selection possible)! | | | | 1. Age 2. Comorbidities 3. Patient’s will 4. Shortage of oxygenators, cannulas, machines 5. ECLS not recommended for COVID-19 by scientific societies 6. Surge of COVID-19 patients (workload to high) 7. Other | | | 80  92  30  6  10  8  12 | 74%  85%  28%  5.5%  9%  7.5%  11% |
|  | Responded: **108** | | Skipped: 154 | |  |  |  |  |  |
|  |  |  |  |  |  |  |  |  |  |
| 5 | Does your center withdraw therapy due to futility? | | | | 1. Yes 2. No | | | 79  30 | 72.5%  27.5% |
|  | Responded: **109** | | Skipped: 153 | |  |  |  |  |  |
|  |  |  |  |  |  |  |  |  |  |
| 6 | Have COVID-19 patients been supported on ECLS for less than 2 weeks? | | | | 1. Yes 2. No | | | 70  34 | 67%  33% |
|  | Responded: **104** | | Skipped: 158 | |  |  |  |  |  |
|  |  |  |  |  |  |  |  |  |  |
| 7 | Have COVID-19 patients been supported on ECLS for more than 4 weeks? | | | | 1. Yes 2. No | | | 52  52 | 50%  50% |
|  | Responded: **104** | | Skipped: 158 | |  |  |  |  |  |
|  |  |  |  |  |  |  |  |  |  |
| 8 | Did your center support COVID-19 patients with more than one ECLS run? | | | | 1. Yes 2. No | | | 47  58 | 45%  55% |
|  | Responded: **105** | | Skipped: 157 | |  |  |  |  |  |
|  |  |  |  |  |  |  |  |  |  |
| 9 | If the decision to discontinue ECLS was made, what there the reasons? | | | | 1. Triage situation 2. Patient’s failure to recover 3. Patient’s assumed or legal representative’s will 4. Shortage of oxygenators, cannulas, machines 5. Lack of staff 6. Prespecified duration met 7. Bleeding (Intracranial Bleeding; ICH) 8. Bleeding (other than ICH) 9. Technical issues 10. Other (including: patient’s recovery: 2) | | | 2  50  5  2  0  1  13  2  1  11 | 2%  53%  5%  2%  0%  1%  14%  2%  1%  12% |
|  | Responded***:  94 (**87**) | | Skipped: 168 | |  |  |  |  |  |
|  |  |  |  |  |  |  |  |  |  |
| 10 | Who decides on withdrawal of treatment in COVID-19? | | | | 1. Treating physician / treating team 2. Treating physician + additional person (e.g. hospital ethical committee, palliative care) 3. Family or next of kin 4. Has to be in agreement with a. or b. and c. 5. Advanced directive / patient’s living will or expressed as witnessed by the family 6. External COVID-19 committee | | | 51  13  4  31  3  0 | 50%  13%  4%  30%  3%  0% |
|  | Responded: **102** | | Skipped: 160 | |  |  |  |  |  |
|  |  |  |  |  |  |  |  |  |  |
| 11 | Was a regulatory authority outside of the hospital involved in how to treat COVID-19 patients? | | | | 1. Yes 2. No | | | 11  96 | 10%  90% |
|  | Responded: **107** | | Skipped: 155 | |  |  |  |  |  |
|  |  |  |  |  |  |  |  |  |  |

***Two participants chose “other”. One was not included since he or she stated that ECLS was not employed at all for COVID-19. The second specified their circuit to be V-AV, which was hence counted as V-VA.**

**** Measured by prothrombin time / activated clotting time**

***** 94 participants answered, but 7 specified reasons for treatment discontinuation without previously choosing “other” as an answer.**

**ECLS** – Extracorporeal membrane oxygenation / **EuroELSO** – European Extracorporeal Life Support Organization / ICU – Intensive care unit / COVID-19 – Coronavirus disease 2019
